# Supplementary material for: Precision stratification of prognostic risk factors associated with outcomes in gestational diabetes mellitus: a systematic review
Source: Commun Med (Lond). 2024 Jan 12;4:9. doi: 10.1038/s43856-023-00427-1 (PMC10786838; doi:10.1038/s43856-023-00427-1)
Supplement: Supplementary file 2 — Supplementary Information [file 43856_2023_427_MOESM2_ESM.pdf]

Title: Precision stratification of prognostic risk factors associated with outcomes in gestational diabetes mellitus: a systematic review

Authors: Zhila Semnani-Azad[1], Romy Gaillard[2], Alice E Hughes[3], Kristen E. Boyle[4], Deirdre K. Tobias[1,5], ADA/EASD PMDI* and Wei Perng[6]
1 Department of Nutrition, Harvard T.H. Chan School of Public Health, Boston, MA, USA
2 Department of Pediatrics, Erasmus MC, University Medical Center, Rotterdam, the Netherlands
3 Faculty of Health and Life Sciences, University of Exeter Medical School, Exeter, United Kingdom
4 Department of Pediatrics and the Lifecourse Epidemiology of Adiposity and Diabetes (LEAD) Center, University of Colorado Anschutz Medical Campus, Aurora, CO, USA
5 Department of Medicine, Brigham and Women's Hospital, Harvard Medical School, Boston, MA, USA
[6] Department of Epidemiology and the Lifecourse Epidemiology of Adiposity and Diabetes (LEAD) Center, University of Colorado Anschutz Medical Campus, Aurora, CO, USA

*A list of authors and their affiliations appear at the end of the paper

Correspondence:
Zhila Semnani-Azad, PhD
E-mail: zsemnaniazad@hsph.harvard.edu
ORCID: 0000-0001-7822-5072

Supplemental Table 1. Diabetes Canada 2018 Clinical Practice Guidelines

Studies of treatment and prevention

**Level 1A**	Systematic overview or meta-analysis of high-quality RCTs<br>a. Comprehensive search for evidence<br>b. Authors avoided bias in selecting articles for inclusion<br>c. Authors assessed each article for validity<br>d. Reports clear conclusions that are supported by the data and appropriate analyses<br>OR<br>Appropriately designed RCT with adequate power to answer the question posed by the investigators<br>a. Patients were randomly allocated to treatment groups<br>b. Follow up at least 80% complete<br>c. Patients and investigators were blinded to the treatment<br>d. Patients were analyzed in the treatment groups to which they were assigned<br>e. The sample size was large enough to detect the outcome of interest
**Level 1B**	Non-randomized clinical trial or cohort study with indisputable results
**Level 2**	RCT or systematic overview that does not meet Level 1 criteria
**Level 3**	Non-randomized clinical trial or cohort study; systematic overview or meta-analysis of level 3 studies
**Level 4**	Other
