# Supplementary material for: Precision stratification of prognostic risk factors associated with outcomes in gestational diabetes mellitus: a systematic review
Source: Commun Med (Lond). 2024 Jan 12;4:9. doi: 10.1038/s43856-023-00427-1 (PMC10786838; doi:10.1038/s43856-023-00427-1)
Supplement: Supplementary file 3 — Description of Additional Supplementary Files [file 43856_2023_427_MOESM3_ESM.pdf]

Description of Additional Supplementary Data

Precision stratification of prognostic risk factors associated with outcomes in gestational diabetes mellitus: a systematic review

Authors: Zhila Semnani-Azad, Romy Gaillard, Alice E Hughes, Kristen E. Boyle,
Deirdre K. Tobias, ADA/EASD PMDI and Wei Perng

File Name: Supplementary Data1_SearchStrategy
Description: Search Strategy

File Name: Supplementary Data2_MaternalOutcomes
Description: Summary of studies included in this systematic review on maternal outcomes.

File Name: Supplementary Data3_OffspringOutcomes
Description: Summary of studies included in this systematic review on offspring outcomes.

File Name: Supplementary Data4_ObsT2DMaternal
Description: Observational studies evaluating prognostic factors associated with incident T2D among women with a history of GDM (n=49).

File Name: Supplementary Data5_RCTT2DMaternal
Description: Randomized controlled trials (RCT) evaluating prognostic factors associated with incident type 2 diabetes (T2D) among women with a history of GDM (n=2).

File Name: Supplementary Data6_ObsCVDMaternal
Description: Observational studies evaluating prognostic factors associated with cardiovascular disease (CVD) among women with a history of GDM (n=6).

File Name: Supplementary Data7_ObsAnthroOffspring
Description: Observational studies evaluating prognostic factors in relation to anthropometric outcomes among offspring exposed to GDM in utero (n=40).

File Name: Supplementary Data8_RCTAnthroOffspring
Description: Randomized control trials evaluating prognostic factors associated with anthropometric outcomes among offspring exposed to GDM in utero (n=5).

File Name: Supplementary Data9_ObsCardiomOffspring
Description: Observational studies evaluating prognostic factors in relation to cardiometabolic outcomes among offspring exposed to GDM in utero (n=14).

File Name: Supplementary Data10_RCTCardiomOffspring
Description: Randomized control trials evaluating prognostic factors associated with cardiometabolic outcomes among offspring exposed to GDM in utero (n=5).
